# Cripto-1 in TNBC

**DOI:** 10.18632/aging.100788

**Published:** 2015-08-15

**Authors:** Nadia P Castro, David S Salomon

**Affiliations:** Tumor Growth Factor Section, Mouse Cancer Genetics Program, National Cancer Institute, Frederick, MD, USA

Among all breast cancer molecular subtypes, triple-negative breast cancer (TNBC) presents the poorest prognosis due to the lack of effective therapeutic options. TNBC, characterized by tumors that do not express estrogen receptor (ER), progesterone receptor (PR), or HER-2 genes, represents approximately 10%–20% of invasive breast cancers and has been associated with African-American race, socio-economic-status, younger age at diagnosis, more advanced disease stage, higher grade, high mitotic indices, a family history of breast cancer and BRCA1 mutations [1]. The search for new therapeutic targets represents an increasing interest to treat this type of breast cancer. The establishment of a clinically relevant mouse model of TNBC is essential to identify and validate genes that might be involved in driving the metastatic cascade, the most lethal aspect of breast cancer, and for potentially screening novel drugs. Moreover, different combinatorial treatments can be tested comparing the effectiveness of various more established and novel chemotherapy modalities since new molecular targets are needed to target this aggressive and lethal type of breast cancer.

Recently we have established a novel spontaneous metastatic model using a feral mouse-derived mammary tumors for studying human TNBC [2]. We performed an in-depth molecular analysis showing that these mouse primary tumors resemble human TNBC both phenotypically and molecularly. Morphologically, primary tumors exhibited evidence of spontaneous epithelial-mesenchymal plasticity, which is a major driver of cancer stem cell generation. Our findings provide evidence that Cripto-1, an embryonic stem cell gene, is a potential factor in promoting mammary tumorigenesis and mesenchymal phenotypes through epithelial mesenchymal transition (EMT) as evidenced by the activation of the Cripto-1 promoter in the EMT areas of the primary tumors. Additionally, our findings reveal a key function for Cripto-1 and that blocking this pathway may offer an alternative treatment strategy for TNBC.

As an interesting note, Tysnes and collaborators have demonstrated that Cripto-1 might represent a novel prognostic marker and therapeutic target in categories of younger patients with glioblastoma suggesting that higher Cripto-1 scores were associated with shorter patient survival [3]. It is possible that Cripto-1 could play a similar role in younger patients with TNBC as compared to other molecular subtypes of breast cancer since higher rates of TNBC have been observed in women who are younger. Moreover, a population-based study of early-onset breast cancer showed a high proportion (approximately 50%) of TNBC among BRCA1 mutations carriers independently of family history (Lee et al 2011). BRCA1 mutations and TNBC status may define a subgroup of patients with breast cancer leading to a more tailored-therapeutic option. BRCA1 mutation carriers are the most common cause of hereditary breast cancer and tend to develop in younger women [4].

Age may represent a prognostic factor for patients in the subtype of TNBC, as a close relationship between age and prognosis is persistent even after accounting for factors as tumor size, grade, and lymph node involvement. These prognostic variables remained independently associated with prognosis after a multivariate survival analysis with 1,732 patients with TNBC showing the unfavorable effect of young age at the diagnosis [5]. One important goal is therefore to generate a more in depth molecular and epidemiological understanding of the genesis and progression of TNBC subtype to elucidate the prevalence of younger patients being more prone to develop TNBC and the biologic aggressiveness for prevention of death. Delineating the biology of TNBC may lead to a more tailored approach for the treatment of this form of breast cancer.
